# Neuroanatomy of Shared Conversational Laughter in Neurodegenerative Disease

**DOI:** 10.3389/fneur.2018.00464

**Published:** 2018-06-15

**Authors:** Peter S. Pressman, Suzanne Shdo, Michaela Simpson, Kuan-Hua Chen, Clinton Mielke, Bruce L. Miller, Katherine P. Rankin, Robert W. Levenson

**Affiliations:** ^1^Division of Behavioral Neurology and Neuropsychiatry, Department of Neurology, University of Colorado Denver, Aurora, CO, United States; ^2^Memory and Aging Center, University of California, San Francisco, San Francisco, CA, United States; ^3^Berkeley Psychophysiology Center, University of California, Berkeley, Berkeley, CA, United States

**Keywords:** laughter, communication, neuroanatomy, empathy, voxel-based morphometry

## Abstract

Perceiving another person's emotional expression often sparks a corresponding signal in the observer. Shared conversational laughter is a familiar example. Prior studies of shared laughter have made use of task-based functional neuroimaging. While these methods offer insight in a controlled setting, the ecological validity of such controlled tasks has limitations. Here, we investigate the neural correlates of shared laughter in patients with one of a variety of neurodegenerative disease syndromes (*N* = 75), including Alzheimer's disease (AD), behavioral variant frontotemporal dementia (bvFTD), right and left temporal variants of semantic dementia (rtvFTD, svPPA), nonfluent/agrammatic primary progressive aphasia (nfvPPA), corticobasal syndrome (CBS), and progressive supranuclear palsy (PSP). Patients were recorded in a brief unrehearsed conversation with a partner (e.g., a friend or family member). Laughter was manually labeled, and an automated system was used to assess the timing of that laughter relative to the partner's laughter. The probability of each participant with neurodegenerative disease laughing during or shortly after his or her partners' laughter was compared to differences in brain morphology using voxel-based morphometry, thresholded based on cluster size and a permutation method and including age, sex, magnet strength, disease-specific atrophy and total intracranial volumes as covariates. While no significant correlations were found at the critical T value, at a corrected voxelwise threshold of *p* < 0.005, a cluster in the left posterior cingulate gyrus demonstrated a trend at *p* = 0.08 (*T* = 4.54). Exploratory analysis with a voxelwise threshold of *p* = 0.001 also suggests involvement of the left precuneus (*T* = 3.91) and right fusiform gyrus (*T* = 3.86). The precuneus has been previously implicated in the detection of socially complex laughter, and the fusiform gyrus has a well-described role in the recognition and processing of others' emotional cues. This study is limited by a relatively small sample size given the number of covariates. While further investigation is needed, these results support our understanding of the neural underpinnings of shared conversational laughter.

## Introduction

Laughter is an ancient and universal emotional expression that often supports social connection (1–3). Laughter primarily occurs in social situations (4), and may reflect recognition of a benign transgression against social expectations or norms rather than anything obviously humorous (5). Laughter occurs an average of five times per 10 min of conversation, usually after fairly mundane, rather than obviously humorous, statements (6). Sometimes, however, social laughter may simply be a nearly automatic response to another's laughter (7). Sharing in another's laughter correlates closely with measures of relationship quality, closeness, and social support (8).

While shared laughter can occur in various situations, e.g., while watching a television show, the emphasis of this study is on conversational laughter. Shared conversational laughter involves both laughter production and perception. Neuroanatomically, laughter production involves brainstem structures including the periaqueductal gray and the upper reticular formation (9), which are under the influence of basal ganglia, hypothalamus, premotor cortices, and basal temporal lobes (3), including the fusiform gyrus (10). Recent studies suggest that networks involved with laughter perception may vary depending on the laughter's nature (11–13). Researchers have characterized laughter in various ways, sometimes demonstrating that different types of laughter have distinctive acoustic properties (9, 11, 14–18). One of the more common distinctions is between “voluntary” and “involuntary” laughter (9, 14, 18), with the former being more internally driven, and the latter being more stimulus driven and externally provoked. The neural substrate supporting perception of voluntary, controlled laughter (as is commonly associated with social interaction) may differ from that supporting perception of involuntary laughter (commonly elicited by tickling). Functional MRI (fMRI) studies have suggested that “social” laughter (e.g., taunting or joyful) activated more medial prefrontal cortex and precuneus compared to tickling laughter, whereas tickling laughter predominantly activated the superior temporal gyrus (11). This may be due to the ambiguity of the social signal, which requires stronger engagement of internal mentalizing by the perceiver (12, 13).

Disorders that impact social interactions can also result in altered laughter patterns. For example, in a recent fMRI study, boys with disruptive behaviors had less mutual laughter and demonstrated less neural reactivity within the supplementary motor and bilateral superior temporal cortices when exposed to social laughter (19). Similarly, altered social dynamics are common among those with neurodegenerative diseases (20). Based on these altered patterns of social interaction, we previously identified different patterns of conversational laughter among some patients with neurodegeneration (21). The purpose of our current study was to investigate the neural correlates of shared conversational laughter during naturally occurring conversation among patients with one of a variety of neurodegenerative illnesses (*N* = 75). Patients with neurodegenerative diseases can serve as a valuable, naturally occurring brain lesion model, in which overlapping regions of volume loss can serve as an indicator of the neural underpinnings of particular behaviors. Each patient with a neurodegenerative disease was seen with a conversational partner in order to assess the frequency of shared laughter.

The neuroanatomy of shared laughter has been studied primarily using task based fMRI studies. While valuable, there are some limitations to the ecological validity of such studies. For example, many studies investigate laughter perception using actor-produced stimuli, which are to some extent all voluntary. Furthermore, classification schema themselves may not account for more ambiguous real-world scenarios. Here, we investigate the neural substrate of shared conversational laughter under conditions of high ecological validity, i.e. semi-naturalistic conversations. While shared or “contagious” laughter has sometimes been described as being less volitional than other laughter types, the complexity of most social interactions may require interpretation and understanding of the other's laughter in order to assess the utility of sharing in that laughter (12, 13), in which case shared conversational laughter may be more controlled and associate more with prefrontal cortex and precuneus. Alternatively, if shared conversational laughter is more involuntary, shared conversational laughter may associate more with the superior temporal lobes (19).

## Materials and methods

### Subjects

Subjects were selected from a standing data repository at University of California, Berkeley's Psychophysiology Laboratory derived from an assessment of emotional functioning that involved multiple emotion-eliciting tasks. The Institutional Review Boards of the University of California, San Francisco, and the University of California, Berkeley, approved the study. All subjects provided informed consent prior to participation.

In order to be included, subjects diagnosed with a neurodegenerative disease had to participate in a conversation (the task of interest) during which their healthy conversational partner laughed at least once. In addition, all subjects had to be part of a group of no fewer than five with a diagnosis of a similar neurological disorder. Only subjects with an MRI scan of the brain within 3 months of the task of interest were included. Seventy-five subjects met these criteria and were included in this research, including Alzheimer's disease (*N* = 11), behavioral variant frontotemporal dementia (bvFTD, *N* = 23), right and left temporal variants of semantic dementia [rtvFTD (*N* = 6), svPPA(*N* = 10)], nonfluent/agrammatic primary progressive aphasia (nfvPPA, *N* = 11), corticobasal syndrome (CBS, *N* = 7), and progressive supranuclear palsy (PSP, *N* = 7). Combined, these diseases relate to bilateral frontal, parietal, and temporal lobar degeneration, offering a wide range of regions that could support shared conversational laughter. Prior to being assessed at Berkeley, all subjects with a neurodegenerative illness underwent a detailed clinical evaluation, including a physical examination and neuropsychological testing. Following this evaluation, their diagnosis was determined by a panel of experts, including neurologists, neuropsychologists, speech pathologists, and nurses.

Alzheimer's disease was established by National Institute on Aging–Alzheimer's Association criteria, and included amnestic, dysexecutive, and behavioral subtypes (22, 23). Consensus criteria were also used to define corticobasal degeneration (24) and progressive supranuclear palsy (25). Primary progressive aphasias (semantic dementia [svPPA] and nfvPPA) were diagnosed using consensus criteria outlined in 2011 (26). In addition, some subjects were diagnosed with the right temporal variant of frontotemporal dementia (rtFTD) by the expert panel by means of available examination and structural magnetic resonance imaging (MRI) data. Those with bvFTD were required to meet 2011 international criteria for inclusion in the study (27, 28). Clinical characteristics of all research groups are detailed in Table 1. Overall, the mean age was 64.3 years, with a mean of 16.8 years of education, mean CDR of 0.8 and mean CDR box of 4.2. Participants were 37.3% female, 92% right-handed, and 94.7% Caucasian. There were no significant demographic differences between groups. Groups did differ in disease severity as assessed by Clinical Dementia Rating (CDR), CDR Box, and Mini Mental State Exam (MMSE) scores, as well as neuropsychological test scores, in the pattern expected for respective diagnoses. While not included in our analysis, basic demographic information was available on 67 out of 75 conversational partners. Spouses or romantic partners comprised 86.6% of conversational partners, with a mean age of 63.6 years (SD 12.3). Conversational partners were 58.2% female. No significant differences in conversational partner demographics were found between groups.

**Table 1:** Demographic and neuropsychological test scores of all included diagnostic groups.

	**AD(*N* = 11)**	**bvFTD(*N* = 23)**	**CBS (*N* = 7)**	**PSP(*N* = 7)**	**nfvPPA(*N* = 11)**	**rtFTD(*N* = 6)**	**svPPA(*N* = 10)**	**Overall(*N* = 75)**
**(A)DEMOGRAPHIC CHARACTERISTICS OF RESEARCH COHORTS**								
Age	62.6 ± 8.4	60.4 ± 8.2	66.3 ± 4.0	68.7 ± 7.2	67.7 ± 11.8	67.7 ± 2.6	64.7 ± 7.4	64.3 ± 8.3
Sex (% Female)	45.5	30.4	42.9	28.6	45.5	50.0	30.0	37.3
Handed (% Right)	81.8	95.6	100	85.7	100	66.7	100	92.0
Ethnicity (% White)	81.8	100	100	85.7	90.9	100	100	94.7
Education	16.2 ± 3.0	16.9 ± 3.6	15.0 ± 0.1	18.7 ± 4.9	17.6 ± 2.0	16.7 ± 2.9	16.5 ± 1.6	16.8 ± 3.1
CDR ^*^	1.2 ± 0.4	1.0 ± 0.5	0.4 ± 0.3	0.6 ± 0.2	0.4 ± 0.3	0.8 ± 0.6	0.4 ± 0.2	0.8 ± 0.5
CDR Box Score ^*^	6.1 ± 2.0	5.7 ± 2.6	3.0 ± 2.5	4.0 ± 2.8	1.5 ± 1.2	4.4 ± 2.2	1.9 ± 1.4	4.2 ± 7.8
**(B)NEUROPSYCHOLOGICAL CHARACTERISTICS OF RESEARCH COHORTS**								
MMSE Score^**^	22.1 ± 4.1	24.7 ± 4.8	26.9 ± 2.1	25.4 ± 2.1	26.9 ± 3.5	26.7 ± 2.8	25.6 ± 3.7	25.2 ± 4.0
CVLT-30 s^**^	3.0 ± 1.9	5.4 ± 1.8	7.1 ± 2.0	6.1 ± 3.0	6.3 ± 1.8	4.6 ± 2.4	2.2 ± 2.3	4.9 ± 2.6
CVLT-10 min^**^	1.6 ± 1.7	4.5 ± 2.4	7.1 ± 1.7	6.0 ± 3.0	5.3 ± 2.6	3.1 ± 3.3	0.9 ± 1.4	4.0 ± 3.0
BNT abbreviated^**^	11.0 ± 4.3	13.2 ± 1.8	14.8 ± 0.5	13.2 ± 1.7	13.3 ± 1.6	9.5 ± 3.8	4.8 ± 3.1	11.4 ± 4.0
Phonemic fluency	9.8 ± 6.1	5.8 ± 5.0	3.1 ± 8.9	3.7 ± 3.5	6.8 ± 6.1	8.0 ± 1.8	7.1 ± 4.5	6.4 ± 5.6
Semantic fluency	7.6 ± 4.6	5.8 ± 5.0	8.5 ± 12.4	12.0 ± 4.4	12.7 ± 8.6	10.6 ± 4.2	5.9 ± 2.8	9.9 ± 6.4
Digit span backwards^*^	2.6 ± 1.0	3.1 ± 1.8	0.1 ± 5.9	3.3 ± 2.5	3.5 ± 1.6	5.0 ± 1.4	3.8 ± 1.8	3.1 ± 2.6
Benson copy ^*^	11.0 ± 4.3	13.6 ± 3.7	7.2 ± 10.5	14.3 ± 2.9	14.1 ± 4.8	14.8 ± 1.0	15.5 ± 1.4	13.1 ± 5.1
Benson recall	2.9 ± 2.6	2.7 ± 1.8	5.3 ± 9.4	6.9 ± 4.3	10.0 ± 5.1	6.0 ± 3.6	7.8 ± 4.0	6.8 ± 5.1
Calculations^*^	3.0 ± 1.3	2.7 ± 1.8	2.1 ± 2.4	2.8 ± 2.3	4.5 ± 1.5	4.7 ± 0.5	4.5 ± 1.6	3.4 ± 1.9
GDS^*^	7.9 ± 5.3	8.2 ± 5.2	7.7 ± 4.0	12.6 ± 7.6	4.2 ± 6.2	2.0 ± 1.7	7.2 ± 5.8	7.2 ± 5.9

*CDR, Clinical Dementia Rating Score; CDR Box, Clinical Dementia Rating Sum of Box Scores; MMSE, Mini-Mental State Exam Score; CVLT, California Verbal Learning Test -II Score; BNT, Boston Naming Test; and GDS, Geriatric Depression Score*.

* *signifies between group differences at p < 0.01*,

** *signifies between group differences at p < 0.001*.

### Task of interest

All assessments were conducted between 2002 and 2016 as part of a broader study of emotional function in neurological disease. Laboratory procedures for obtaining samples of conversations between patients and caregivers were derived from well-established methods (29). Each patient and their conversational partner, usually a family member or close friend, was instructed to discuss a mutually selected topic of continuing disagreement in their relationship in order to evoke emotional reactivity. Each conversation lasted between 10 and 15 min. Audio recordings of the conversations were obtained using unidirectional lavaliere microphones attached to each conversationalist.

### Acoustic labeling

The audio from conversations was saved in WAV format. All speech and nonspeech sounds (such as laughter) were manually labeled in Praat, an acoustic analysis program (30, 31) by trained research assistants based on their own judgment of what constituted laughter (Supplementary Figure 1).

### Measure of interest

Based on a previously described classification of conversational laughter (32), when a laugh was detected, it was categorized as being related to the partner's laughter if the laugh occurred during or within 3 s following the partner's laughter. Laughter was automatically categorized via a script written for Stata 13.0. The automated categorization of the first 100 laughs collected was compared with a human rater, with 100 percent agreement. All subsequent laughs were categorized using the automated procedure. For each participant, the probability of laughing relative to his or her partner's laughter was calculated as being the number of laughs relating to partner laughter divided by the total number of times the partner laughed.

### MRI acquisition

All participants with a neurodegenerative disease underwent a structural MRI scan on a 1.5, 3, or 4T Magnetom VISION system (Siemens Inc. Iselin, N.J.) within 3 months of the conversation. T1-weighted whole brain images were obtained using a volumetric magnetization prepared rapid gradient echo MRI sequence (MPRAGE, TR/TE/TI = 10/4/300 ms) with 15° flip angle, coronal orientation perpendicular to the double spin echo sequence, 1.0 × 1.0 mm^2^ in-plane resolution and 1.5 mm slab. Scans were visually inspected for excessive movement prior to inclusion.

### Image pre-processing

Voxel-based morphometry (VBM) preprocessing and analysis were performed using the VBM8 toolbox (http://dbm.neuro.uni-jena.de/vbm/) and Statistical Parametric Mapping 8 (SPM8) software (http://www.fil.ion.ucl.ac.uk/spm/software/spm8/). Following bias-correction and tissue-classifications, segmented images were normalized to MNI space with a 1.0 mm cubic resolution using affine and nonlinear transformations via the diffeomorphic anatomical registration through exponentiated lie algebra (DARTEL) method (33, 34). DARTEL was also used to create a customized template based on 300 older healthy controls. Default parameters of the VBM8 toolbox were used in all preprocessing steps except for adding a previously described light clean-up procedure in the morphological filtering step (35). Spatially normalized, segmented, and modulated gray matter images were then smoothed with an 8-mm FWHM isotropic Gaussian kernel.

### Voxel-based morphometry analysis

We used multiple regression design analyses to correlate the probability of a patient with neurodegeneration laughing relative to their partner's conversational laughter with gray matter atrophy across the sample of 75 participants. Age, gender, magnet strength, MMSE (as a proxy for disease severity), and TIV were used as standard covariates. The Geriatric Depression Scale (GDS) was considered as a potential covariate, but ultimately disregarded as GDS did not correlate with the probability of laughing in relation to the partner's laughter, i.e., the primary variable of interest (*p* = 0.35). Because VBM of neurodegenerative disease can lead to co-atrophy artifact, wherein regions unrelated to a task appear as statistically significantly related due to disease specific co-atrophy patterns, each diagnosis was also parameterized and entered as a confounding covariate in the analysis. This helps to ensure that regions of atrophy are associated with laughter probability only if those associations are present in more than one diagnostic group (36, 37), improving generalizability of results.

A voxel-wise threshold of *p* < 0.005 was used to threshold the resulting statistical parametric map, which was then corrected for multiple comparisons at *p* < 0.05 based on cluster extent and a custom-fit error distribution based on 1,000 data permutations (35). This permutation analysis also helps correct for deviations from parametric data distributions, that was required given a zero-inflated distribution of our data (38). Because we recognized that our sample size was relatively small given the high number of covariates involved, we permitted exploratory analysis at an unadjusted threshold of *p* < 0.001 should results indicate a region of marginal non-significance at a *p* < 0.10. SPM T-maps were superimposed on the Montreal Neurological Institute (MNI) single subject brain template using automated anatomical labeling included in the MRIcron software package.

## Results

Across all groups in the 10-min conversation, the median number of laughs was 2, with a range of 0–38. Further information on laughter by group is listed in Table 2. The counts and probabilities are generally zero-inflated, with wide variation between individuals. Neither negative binomial regression nor zero-inflated Poisson (correcting for zero-inflation) found differences between groups in any laugh-related measure.

**Table 2:** Laughter characteristics by group.

	**AD(*N* = 11)**	**bvFTD (*N* = 23)**	**CBS (*N* = 7)**	**PSP (*N* = 7)**	**nfvPPA (*N* = 11)**	**rtFTD (*N* = 6)**	**svPPA (*N* = 10)**	**Overall (*N* = 75)**
Total laughter	3 ± 3.6	4.2 ± 9.0	2.9 ± 3.9	4 ± 4.6	10.4 ± 7.3	2.7 ± 4.1	3.9 ± 3.6	4.6 ± 6.7
	**2 (0–12)**	**2 (0–38)**	**1 (0–11)**	**4 (0–13)**	**7 (2–23)**	**1 (0–11)**	**2.5 (0–10)**	**2 (0–38)**
Laughs related to partner's laughter	0.6 ± 1.0	0.7 ± 1.7	0.3 ± 0.5	1.1 ± 1.9	1.7 ± 1.6	0.3 ± 0.5	0.2 ± 0.4	0.76 ± 1.4
	**0 (0–3)**	**0 (0–6)**	**0 (0–1)**	**0 (0–5)**	**2 (2–4)**	**0 (0–1)**	**0 (0–1)**	**0 (0–6)**
Probability of laughing if partner laughs	13.2 ± 22.2	9.3 ± 22.4	5.7 ± 12.4	25.8 ± 38.8	29.2 ± 31.6	6.9 ± 13.4	11.2 ± 31.4	14.1 ± 26.1
	**0 (0–60)**	**0 (0–100)**	**0 (0–33.3)**	**0 (0–100)**	**30 (0–100)**	**0 (0–33.3)**	**0 (0–100)**	**0 (0–100**)

*Laugh counts and probabilities between populations. Mean and standard deviation are on top, followed by median and range on bottom in bold. Note zero-inflation frequently dropping the median below the mean. Due to high variance, no significant differences were found between any measures after assessing with models incorporating zero-inflation*.

**Figure 1:**
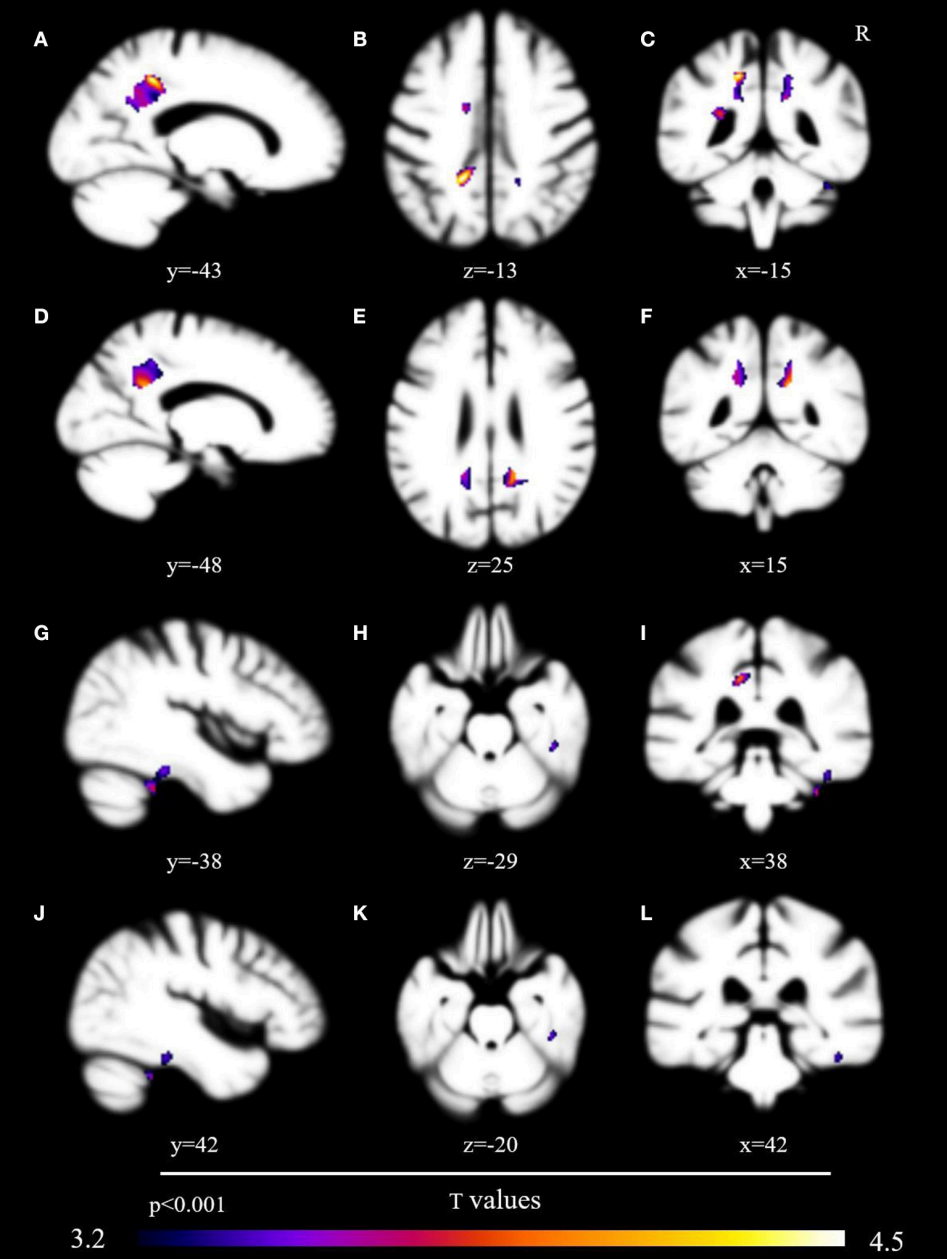
Brain volumetric correlates with probability of sharing in conversational laughter. Brain regions associated via voxel-based morphometry with the probability of sharing in a conversational partner's laughter, presented uncorrected at *p* < 0.001 after analysis with the permutation method. Regions include the left posterior cingulate gyrus **(A–C)**, precuneus [images **(A,C–F,I)**], right fusiform gyrus [images **(G–L)**], and left supplementary cortex [images **(B,I)**].

No voxels met the critical T value of 5.84. At a corrected voxelwise threshold of *p* < 0.005, a cluster in the left posterior cingulate gyrus demonstrated marginal non-significance at *p* = 0.08 (*T* = 4.54). We found decreased gray matter at a threshold of *p* < 0.001 in nine areas: left posterior cingulate gyrus (*T* = 3.97), left precuneus (*T* = 3.90), right fusiform gyrus (*T* = 3.87), right cerebellum (*T* = 3.63), left middle cingulate (*T* = 3.47), left supplementary motor cortex (*T* = 3.37), right posterior cingulate cortex (*T* = 3.36), left anterior cingulate cortex (*T* = 3.36), and right inferior temporal gyrus (*T* = 3.28) (Table 3, Figure 1).

**Table 3:** Neuroimaging correlates between volumes and probability of sharing in laughter.

**maxT**	**Region of interest**	**x**	**y**	**z**
3.97	Left posterior cingulate gyrus	−9	−37	37
3.91	Left precuneus	−10	−41	40
3.87	Right fusiform gyrus	37	−37	−28
3.63	Right cerebellum exterior	34	−34	−31
3.48	Left middle cingulate gyrus	−10	−4	40
3.37	Left supplementary motor cortex	−10	−5	41
3.37	Right posterior cingulate gyrus	13	−47	32
3.36	Left anterior cingulate gyrus	−7	33	−3
3.28	Right inferior temporal gyrus	43	−31	−19

*T-value list for laughter correlates. List of all regions with a positive correlation between the brain volume and probability of laughing during or shortly after the healthy conversational partner's laugh at significance level p < 0.001*.

## Discussion

While they should be considered exploratory, our findings suggest that the probability of laughing due to someone else's laughter in everyday conversation may positively correlate with brain volumes in the posterior cingulate cortex. Additional exploratory analysis implicates the precuneus, right fusiform gyrus, left supplementary motor cortex, and left anterior cingulate, all of which have been previously been implicated with laughter in the past.

Our findings lend support to existing theories of the neural substrate of shared laughter using a methodology with greater ecological validity. Although our findings should be interpreted with caution, a potential role for the implicated regions has previously been supported by functional neuroimaging. For example, among healthy research participants Wildgruber and colleagues found the posterior cingulate and precuneus to be highly involved in the perception of socially complex laughter, such as that which conveys joy or taunting (12). These brain regions have been shown to be involved with networks that support mentalizing processes and theory of mind (39). This suggests that in most conversation, laughing even in response to another's laughter is not necessarily automatic, but rather depends on the evaluation of the laughter's context and social implications prior to a response.

Our results also suggest involvement of the right fusiform gyrus in mutual laughter. The right fusiform gyrus has been widely implicated in the processes of facial and emotional recognition, but also in the more automatic generation of laughter when electrically stimulated (10). Others who have described correlations between laughter perception and the fusiform gyrus have suggested that visual imagery of laughing faces may be elicited by the laughter (40–42). While most of these studies investigated the acoustics of laughter alone, our participants were able to see their partner's face, which may account for some of this correlation.

Our exploratory results also implicate the anterior and middle cingulate gyrus and supplementary motor area (SMA). The cingulate and supplementary motor cortices have been previously associated with production of involuntary vocalizations, such as those associated with emotions (43). The SMA and pre-SMA have previously been correlated with listening to emotional signals, such as laughter, particularly when emotionally complex (13), and while producing related facial movement (44).

Contrary to what one would predict for an anatomy of predominantly involuntary laughter, we did not find involvement of the superior temporal lobes. Similarly, while some studies have correlated shared laughter to the anterior insula, we did not find this in our research. This may be due in part to a methodological limitation. As we co-varied for each disease type, regions that predominantly atrophied with only one disorder were essentially removed from the analysis. For example, anterior insular degeneration is common in bvFTD (20). Due to the relative specificity of this region to that disorder, including bvFTD as a covariate could essentially remove it from inclusion in our findings. Nevertheless, superior temporal lobar degeneration can be involved in a wider array of neurodegenerative diseases. We believe that the relative lack of their involvement here suggests a relatively small role compared to brain regions that mediate a more volitional sharing of conversational laughter.

### Strengths and weaknesses

This study is limited by a relatively small sample size given the number of covariates. Thus, results are exploratory, and should be interpreted accordingly. The covariate structure of this VBM was designed to avoid co-atrophy artifact—as discussed, however, this approach may fail to identify regions truly related to expression scores that are atrophied in only one diagnostic group. While this approach does increase plausible generalizability of results by ensuring correlations are present in more than one patient group, we only studied scans from patients with neurodegeneration, not healthy individuals. Other weaknesses include a paucity of information on patients' conversational partners. Furthermore, our experimental design and labeling system only captures audible laughter and does not easily permit exploration of causes of an individual's laughter. For this reason, we could not discern possible contributions of laughter due to phenomena like pseudobulbar affect, which would have a different contributing anatomy. The instruction to discuss a topic of mutual disagreement may have reduced our chances of eliciting laughter—other instructions may have increased the number of laughs in this study and lent even greater ecological validity to our results. This low level of laughter overall has been previously described using the same task. As in that publication, nfvPPA stands out from other groups in conversational laughter production, which may represent a paralinguistic method of social connection to compensate for an often-frustrating apraxia of speech (21). Further studies in other samples would be necessary to confirm and help explain this behavior.

Despite those weaknesses, our results are consistent with previous research on brain regions involved with shared laughter. The use of a task with high ecological validity sheds further light on structures that are most likely to be relevant to sharing in everyday conversational laughter. Our findings support theories that envision this interaction as being less automatic than the response commonly elicited in more narrowly defined task-based designs. In everyday interaction, shared laughter likely depends on a degree of internalization and processing of other socially relevant information.

### Future directions

As with previous studies of laughter in neurodegenerative disease, there is substantial variance in laugh behavior. Future studies may reduce this variance by focusing on longitudinal changes in laughter within individuals. Because shared conversational laughter is associated with higher measures of relationship quality, future studies should also consider exploring how changes in laugh behavior impact the relationship between patients and caregivers coping with neurodegenerative disease, as well as influence caregiver perceptions of behavioral manifestation of neurodegenerative disease as rated on standardized questionnaires and directly reported to physicians.

## Conclusions

A network including the cingulate cortex, precuneus, fusiform gyrus, and supplementary motor area likely mediates the probability of someone sharing in another person's conversational laughter. These findings are in accordance with and offer further ecological validity to prior models describing the perception and sharing in socially complex laughter.

## Author contributions

PP, KR, and RL Study conception and design. PP, MS, K-HC, SS, BM, KR, and RL Acquisition of data. PP and CM Analysis and interpretation of data. PP Drafting of manuscript. PP, MS, CM, K-HC, SS, BM, KR, and RL Critical revision.

### Conflict of interest statement

The authors declare that the research was conducted in the absence of any commercial or financial relationships that could be construed as a potential conflict of interest. The reviewer JB declared a past co-authorship with one of the authors BM to the handling Editor.

## Supplementary material

Supplementary Figure 1 A sample praat grid labeling laughter. A depiction of how laughs were labeled for each speaker using Praat. Each speaker was represented by one tier for speech (< sp>), and another for nonspeech sounds (e.g., < laugh>). The sample represents an instance where Speaker 2 laughed following speech by Speaker 1. Speaker 1 then joined in that laughter before resuming speech.
