# Supplementary figures and images for: Neuroanatomy of Shared Conversational Laughter in Neurodegenerative Disease
Source: Front Neurol. 2018 Jun 15;9:464. doi: 10.3389/fneur.2018.00464 (PMC6013725; doi:10.3389/fneur.2018.00464)

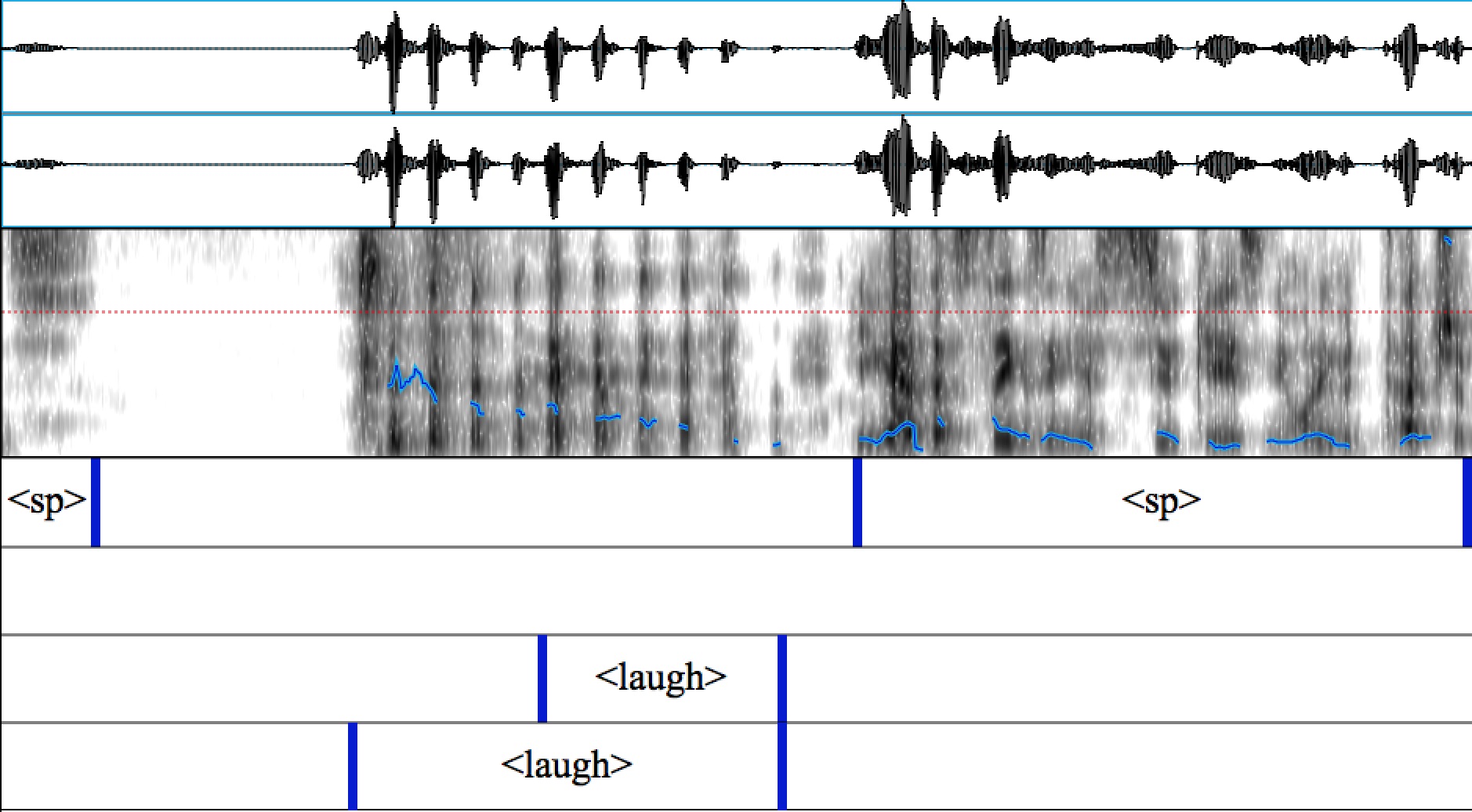

Supplement: Supplementary Figure 1 — A sample praat grid labeling laughter. A depiction of how laughs were labeled for each speaker using Praat. Each speaker was represented by one tier for speech (< sp>), and another for nonspeech sounds (e.g., < laugh>). The sample represents an instance where Speaker 2 laughed following speech by Speaker 1. Speaker 1 then joined in that laughter before resuming speech. [file Image_1.JPEG]
